# Lower prevalence of *Blastocystis* spp. and *Dientamoeba fragilis* in ulcerative colitis and association with disease activity

**DOI:** 10.1007/s10096-026-05429-0

**Published:** 2026-02-23

**Authors:** Mèlanie Valentina Bénard, Rens Zonneveld, Patricia Broekhuizen-van Haaften, Ellen M. S. Wentink-Bonnema, Sebastien Matamoros, Cyriel Y. Ponsioen

**Affiliations:** 1 Department of Gastroenterology and Hepatology, Amsterdam UMC, Amsterdam, the Netherlands; 2 Department of Medical Microbiology and Infection Prevention, Amsterdam UMC, Amsterdam, the Netherlands

## Abstract

**Aim:**

To evaluate the prevalence of intestinal protozoan parasites *Blastocystis spp.* and *Dientamoeba fragilis* in ulcerative colitis (UC) patients and healthy controls, and to assess their association with UC disease activity.

**Methods:**

Fecal samples were collected from 103 UC patients and 95 healthy volunteers during the screening of the TURN2 trial, a randomized controlled trial on the efficacy of anoxic-prepared fecal microbiota transplantation (FMT) in active UC. The presence and load of protozoa were assessed by microscopy and PCR. Disease activity was assessed using the Simple Clinical Colitis Activity Index (SCCAI), Partial Mayo Score and fecal calprotectin (FCP).

**Results:**

The prevalence of *Blastocystis* spp. was significantly higher in healthy controls (33% by microscopy and 34% by PCR, respectively) compared to UC patients (6% by both methods, *p* < 0.001). Similarly, *D. fragilis* was more common in healthy controls (34% by microscopy and 37% by PCR) than UC patients (9% and 12% respectively, both *p* < 0.001). Microscopic detection of *D. fragilis* was significantly associated with lower disease activity, as measured by the Simple Clinical Colitis Activity Index (median [IQR]: 4 [4] vs. 6 [4], *p* = 0.023) and the Partial Mayo Score (median [IQR]: 4 [2] vs. 6 [2], *p* = 0.009). Amongst the *D. fragilis* positive samples, higher *D. fragilis* loads, as assessed by either microscopy or PCR, were significantly associated with lower FCP levels (*p* = 0.010 and *p* = 0.040). PCR detection of *D. fragilis* showed similar but non-significant associations with disease activity scores. No associations were found between *Blastocystis spp.* and disease activity.

**Conclusion:**

Our findings show a higher prevalence of *Blastocystis* spp. and *D. fragilis* in healthy individuals compared to UC patients, challenging their pathogenicity in the context of UC and in general. The reported association between *D. fragilis* presence and load and lower UC disease activity warrants further investigation in larger cohorts.

## Introduction

Intestinal protozoa, particularly *Blastocystis* spp. and *Dientamoeba fragilis*, are commonly found in the fecal microbiotas of healthy individuals [1, 2]. Their pathogenic potential has been widely debated since their discovery [3–5]. Increasing evidence shows that the presence of *Blastocystis* spp. is associated with higher bacterial alpha-diversity, which is considered a marker of gut health, and distinct microbial profiles in the gut [2, 6, 7]. Interestingly, studies have shown that patients with inflammatory bowel disease (IBD), including ulcerative colitis (UC), tend to have a lower prevalence of *Blastocystis* spp., particularly described during active disease phases [2, 8, 9]. This raises the question of whether disease activity influences the prevalence of *Blastocystis* spp., or whether these protozoa, along with their associated bacterial profiles, could play a role in modulating disease activity in UC.

While *Blastocystis* spp. has been more widely reported in the context of UC, data on the presence of *D. fragilis* in UC patients are scarce. Only two small-scale studies have investigated this, with one reporting a lower prevalence of *D. fragilis* in active UC, [9] while a previous study from our center did not find significant differences between active UC patients and healthy controls [8]. Given the limited data available, further exploration of the role of *D. fragilis* in UC could help clarify its potential involvement.

The primary aim of this study was to compare the prevalence and load of these intestinal protozoa between healthy volunteers and a larger cohort of UC patients with varying disease activity. Additionally, we explored the association between presence of these protozoa and clinical disease parameters, including disease activity scores, to gain further insight into their potential role in UC.

## Methods

### UC patients

Fecal samples from UC patients were collected for the present study between May 2020 and August 2023, during the screening process of the TURN2 trial, a multi-center randomized controlled trial evaluating the efficacy of anoxic-prepared donor fecal microbiota transplantation (FMT) compared to autologous FMT in active ulcerative colitis (ClinicalTrials.gov: NCT05998213). The screening process began with questionnaires, blood tests, and fecal analyses to assess active disease and evaluate exclusion criteria. The questionnaires included the Simple Clinical Colitis Activity Index (SCCAI) and stool diaries were used to determine components of the Partial Mayo Score. Fecal investigations included measurements of fecal calprotectin levels and testing for active infections with pathogens such as *Salmonella*, *Shigella*, and *Clostridium difficile*. Positive microscopy and/or PCR for protozoa, specifically high microscopic amounts of *Blastocystis* spp., and/or microscopic or molecular evidence of *D.* fragilis or *Giardia lamblia*, were also exclusion criteria. If the initial screening indicated signs of active disease and no exclusion criteria were met, a sigmoidoscopy was performed as a second step to assess the extent of disease and the endoscopic Mayo Score. Patients were allowed to use a stable dose of thiopurines and/or mesalamines. Corticosteroid use was permitted at doses < 15 mg/day, with mandatory tapering during the study. The use of biologics and Janus kinase (JAK) inhibitors was not allowed within two months prior to study participation. Inclusion criteria for active disease for participation in the clinical trial were a Full Mayo Score of 5–9 with an endoscopic Mayo Score of ≥ 2 in the rectum and/or sigmoid. For the purpose of this study, all screened patients were included in the evaluation of disease activity in relation to the presence of protozoa. The study protocol was approved by the Medical Ethical Committee of the Amsterdam UMC in the Netherlands (reference number 2018_057).

## Healthy volunteers

Fecal samples from healthy volunteers were obtained from individuals who were screened as potential fecal donors for the TURN2 trial. The screening process included a prescreening interview and an extensive questionnaire assessing risk factors for (infectious) diseases and microbiota-altering factors. Only those who successfully completed this questionnaire proceeded to further screening, starting with microscopy and PCR testing for intestinal parasites. Detailed donor selection criteria and procedures have been described previously [1]. Key inclusion criteria were age 18–55 years, a normal BMI (18.5–26), and no medication use (including antibiotics in the past four weeks). Exclusion criteria included smoking, use of pre- and probiotics, gastrointestinal symptoms, a history of or current clinical evidence of gastrointestinal, autoimmune, psychiatric, or neurological disorders, and risk factors for transmissible diseases.

## Testing for intestinal parasites

The procedures for detecting protozoa were performed identically for UC patients and donor samples, and by the same experienced technicians. Fresh fecal samples were collected in two tubes: one containing unfixed feces and the other containing Sodium Acetate Formaldehyde (SAF) fixative. Unfixed fecal samples were concentrated according to the method by Allen and Ridley et al. [10] and then used for microscopy for the detection of protozoan cysts and eggs of intestinal helminths. Real time PCR was performed on unfixed feces for *Blastocystis* spp., *Cryptosporidium* spp., *D. fragilis*, *Entamoeba histolytica*, and *Giardia lamblia*. The SAF-fixed fecal samples were used for microscopic examination of the vegetative stages of intestinal protozoa. These samples were stained using Kop-Color stain (Elitech, Puteaux) for *Blastocystis* spp. and Chlorazol black stain for *D. fragilis* and examined under conventional light microscopy. The presence and load of *Blastocystis* spp. and *D. fragilis* were assessed, with the load categorized as ‘sporadic’, ‘few’, ‘some’, ‘moderate’, or ‘many’.

## Statistical evaluation

Data were expressed as the median and interquartile range (IQR), as normality assumptions were not met. The prevalence of *Blastocystis* spp. and *D. fragilis* was compared between UC patients and healthy controls using Chi-square tests for expected cell sizes > 5, and Fisher’s exact test for smaller cell sizes. The association between the presence of protozoa and the continuous SCCAI, Partial Mayo Scores, and fecal calprotectin was evaluated using the Mann-Whitney U test. The SCCAI was categorized into active and non-active disease using a cutoff of 5, as established in the literature, and the association with protozoa presence was analyzed using the Chi-square test. Spearman’s rank correlation was used to assess the relationship between protozoa load (both microscopy and Ct-values) and fecal calprotectin levels, as well as between load and clinical disease activity (SCCAI and Partial Mayo Scores). The distribution of *D. fragilis* load categories between the high and low FCP groups was compared using Fisher’s exact test, due to small sample sizes in some categories. Finally, a linear regression model was built to assess whether potential confounders, including five medication groups (i.e., mesalamines, corticosteroids, thiopurines, proton pump inhibitors, and iron supplements), BMI, age, and sex, influenced the observed relationships between protozoa presence and disease activity as assessed with SCCAI and Partial Mayo. Statistical analyses were conducted using SPSS version 28.0 software (IBM Corp., Armonk, NY, USA). A p-value of < 0.05 was considered statistically significant.

## Ethics approval

This sub-analysis of the TURN2 trial (ClinicalTrials.gov: NCT05998213, registration date 23 August 2023) was approved by the Medical Ethics Review Committee of Amsterdam UMC and conducted in accordance with the Declaration of Helsinki. All participants provided written informed consent.

## Results

In total, 95 healthy volunteers and 103 UC patients were evaluated for this study. Gender was evenly distributed across both groups. Healthy volunteers were younger than the evaluated UC patients (*p* < 0.001) and had a lower BMI (*p* < 0.001). None of the healthy volunteers were active smokers, as smoking was an exclusion criterion for donors. In contrast, 9 UC patients (8.7%) were active smokers. An overview of the study population characteristics and medication use at inclusion is shown in Table 1.

**Table 1:** Baseline characteristics of study population

	Healthy controls*N* = 95	UC Patients*N* = 103	*p*-value
Gender male, *n* (%)	37 (38.9)	45 (43.7)	0.564
Age, years (median, IQR)	29 (11.0)	40 (24.0)	< 0.001
BMI (median, IQR)	21.8 (3.2)	23.4 (4.4)	< 0.001
Active smoker	0 (0)	9 (8.7)	< 0.001
Disease duration in years (median, IQR)		8.3 (11.6)	-
History of disease extent, proctitis Montreal E1		12 (11.7)	-
History of disease extent, left-sided colitis Montreal E2		39 (37.9)	-
History of disease extent, pancolitis Montreal E3		49 (47.6)	-
Mesalamines (oral)		67 (65)	-
Corticosteroids, topical or oral		18 (17.5)	-
Thiopurines		12 (11.7)	-
Proton pump inhibitors		9 (8.7)	-
Iron supplements		3 (2.9)	-

### Prevalence of *Blastocystis* spp. and *Dientamoeba fragilis* in healthy volunteers and UC patients

A significant difference in the detection of intestinal protozoa was observed between healthy controls and UC patients (Table 2). The prevalence of *Blastocystis* spp. was significantly higher in healthy controls, both observed with microscopy (32.6% vs. 5.8%, *p* < 0.001) as detected by PCR (33.7% vs. 5.8%, *p* < 0.001). Similarly, *D. fragilis* was more frequently detected in healthy controls, with microscopy showing a prevalence of 33.7% in healthy controls compared to 8.7% in UC patients (*p* < 0.001), while PCR detection yielded slightly higher prevalence rates (36.8% vs. 11.7%, *p* < 0.001), reflecting its higher sensitivity. Furthermore, co-infection of *Blastocystis* spp. and *D. fragilis* was significantly more common in healthy controls, both by microscopy (17.9% vs. 1.9%, *p* < 0.001) and PCR (20.0% vs. 1.9%, *p* < 0.001).

**Table 2:** Prevalence of *Blastocystis* spp. and *Dientamoeba fragilis* in UC patients and healthy volunteers

	Healthy controls*n* = 95	UC patients*n* = 103	*p*-value
*Blastocystis spp. positive microscopy, n (%)*	31 (32.6)	6 (5.8)	< 0.001
*Blastocystis* spp. detected by PCR, n (%)	32 *(33.7)*	6 *(5.8)*	< 0.001
*Dientamoeba fragilis* positive microscopy, n (%)	32 *(33.7)*	9 *(8.7)*	< 0.001
*Dientamoeba fragilis* detected by PCR, n (%)	35 *(36.8)*	12 *(11.7)*	< 0.001
*Dientamoeba fragilis* and *Blastocystis* spp. positive microscopy, n (%)	17 *(17.9)*	2 *(1.9)*	< 0.001
*Dientamoeba fragilis* and *Blastocystis* spp. PCR positive, n (%)	19 *(20.0)*	2 *(1.9)*	< 0.001

### *Blastocystis* spp. and *Dientamoeba fragilis* in relation to UC disease activity

No significant associations were found for *Blastocystis spp.* and disease activity markers.

**Fig. 1:**
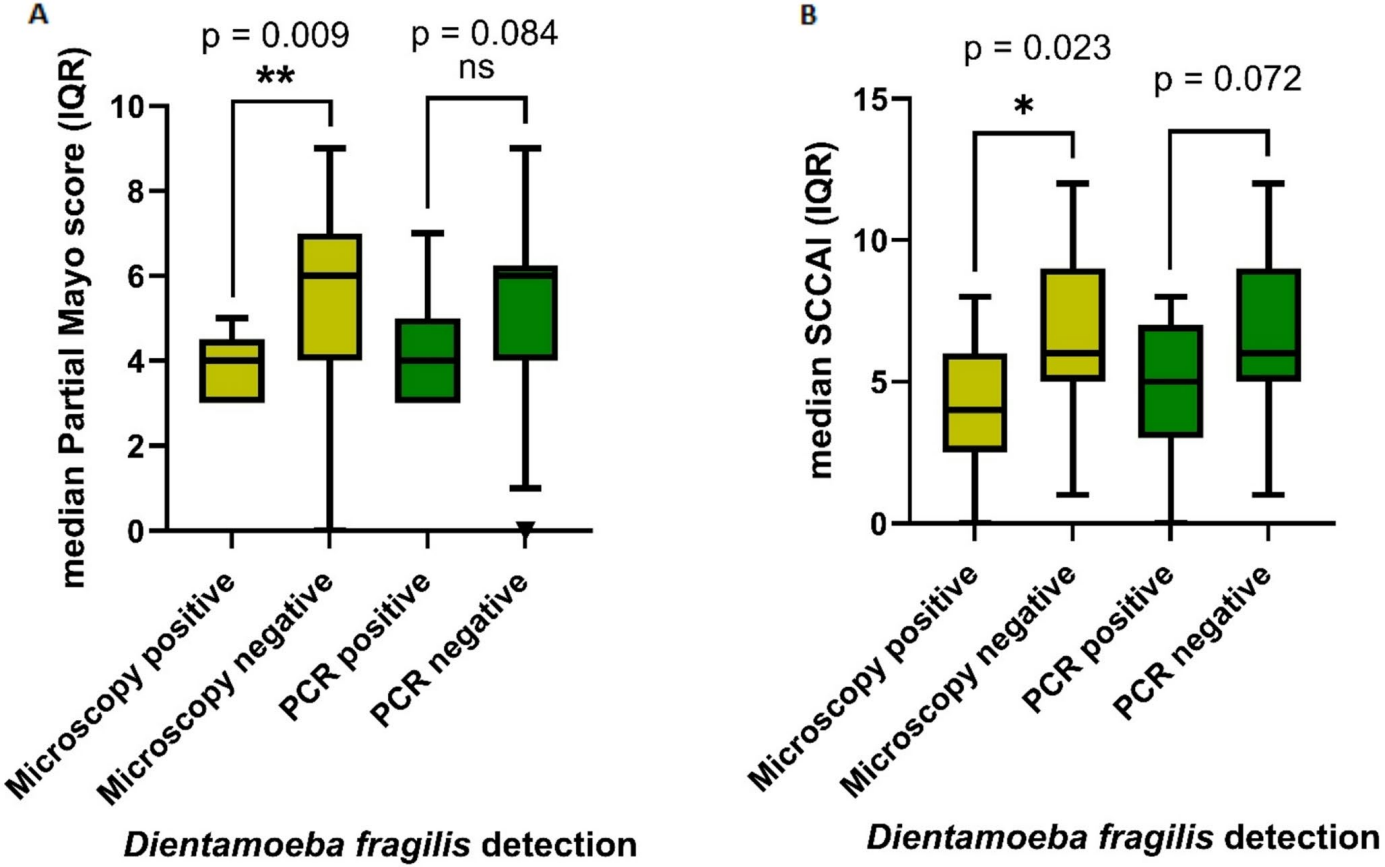
*Dientamoeba fragilis* detection by microscopy and PCR, in relation to SCCAI scores (**A**) and Partial Mayo scores (**B**). Median and interquartile ranges are shown. * *p* < 0.05, ** *p* < 0.01

In contrast, *D. fragilis* detection by microscopy was significantly associated with lower disease activity, as measured by SCCAI and Partial Mayo Scores. Patients in whom *D. fragilis* was detected microscopically had significantly lower SCCAI scores compared to those in whom it was not detected (Fig. 1A, median [IQR]: 4 [4] vs. 6 [4], *p* = 0.023). Similarly, positive microscopy for *D. fragilis* presence was associated with lower Partial Mayo Scores (Fig. 1B, median [IQR]: 4 [2] vs. 6 [2], *p* = 0.009). Consistently, when categorizing patients based on disease activity (SCCAI ≥ 5 indicating active disease), *D. fragilis* detection by microscopy was more frequent in patients with non-active disease than in those with active disease (*p* = 0.036). The association between microscopic *D. fragilis* presence and SCCAI and Partial Mayo scores remained significant in multivariate logistic regression after adjusting for medication use (5 groups), BMI, age, and gender.

Detection of *D. fragilis* with PCR showed similar but non-significant associations in relation to both SCCAI (median [IQR]: 5 [4] vs. 6 [4], *p* = 0.072) and Partial Mayo Scores (median [IQR]: 4 [2] vs. 6 [2], *p* = 0.084).

### Association between *D. fragilis* load and fecal calprotectin

Across the entire cohort, microscopic detection of *D. fragilis* was not associated with FCP levels (*p* = 0.218). However, within the microscopy-positive cases, a significant correlation was observed between FCP and the load of *D. fragilis*, as assessed by both microscopy and Ct-values. Specifically, microscopic load of *D. fragilis* was negatively correlated with FCP (ρ = -0.798, *p* = 0.010), indicating that higher *D. fragilis* loads were associated with lower FCP levels. Similarly, Ct-values were positively correlated with FCP (ρ = 0.599, *p* = 0.040), with lower Ct-values (which indicate a higher *D. fragilis* load) associated with lower FCP levels.

When categorizing fecal calprotectin into elevated (> 250 µg) and non-elevated (< 250 µg) groups, *D. fragilis* microscopic load was differently distributed between the high and low FCP groups (Fisher’s exact, p = 0.036). Specifically, the load category ‘many’ was only detected in the non-elevated FCP group, while the lower categories (‘sporadic’ to ‘moderate’) were more frequently detected in patients with elevated FCP. No significant correlations were observed between the microscopic load of *D. fragilis* and clinical SCCAI and Partial Mayo Scores.

## Discussion

In this study, we investigated the prevalence of the protozoa *Blastocystis spp*. and *D. fragilis* in healthy volunteers and UC patients. Among asymptomatic volunteers, these protozoa were frequently detected (*Blastocystis* spp.: 34%; *D. fragilis*: 38%), with significantly higher prevalence compared to UC patients, in whom they were detected in only 6% and 12% of cases, respectively. Additionally, an association was observed between the presence of *D. fragilis* and lower UC disease activity scores.

For *Blastocystis* spp., no association with disease activity was observed. In our cohort only six UC patients tested positive for *Blastocystis* spp., limiting the ability to detect a potential association with disease activity. However, these findings are consistent with previous UC cohort studies [11, 12] in which no significant difference in *Blastocystis* prevalence was reported between active disease and remission, suggesting reduced prevalence in UC patients regardless of disease activity. Research on *D. fragilis* in the context of UC is limited. A previous study from our center similarly reported a lower prevalence of *D. fragilis* in active UC patients compared to healthy individuals, although the difference was not statistically significant [8]. In addition, one small-scale study by Petersen et al. [9] reported that *D. fragilis* was more frequently detected in patients with inactive UC than in those with active disease, which aligns with current findings.

The reduced prevalence of these protozoa in UC compared to healthy controls may reflect an altered disease-associated gut microbiota or environmental factors that create conditions less favorable for their survival. Increasing evidence now strongly associates the presence of *Blastocystis* spp. with higher alpha-diversity and distinct bacterial species and profiles, [2, 6, 7] including *Clostridia*, [7] *Prevotella copri*, [6] and Prevotella-driven profiles, [6, 13] as well as negative correlations with *Enterobacteriaceae* [7] and *Bacteroides*-dominated profiles [13]. Notably, associations between specific bacterial types and *Blastocystis* spp. subtypes appear to be stronger than those with host variables [2]. Correspondingly, UC is associated with lower alpha-diversity and altered microbial profiles, including a decrease of Bacillota (formerly known as Firmicutes), comprising Clostridia [14, 15]. Less data is available on microbial characteristics in the presence of *D. fragilis*, but similar to *Blastocystis* spp., higher bacterial alpha-diversity in *D. fragilis* positive samples has been reported [16]. Furthermore, both *Blastocystis* spp. and *D. fragilis* were associated with a relative clustering of bacterial profiles, regardless of symptoms, in a combined analysis of 419 fecal samples of IBS-patients, asymptomatic participants, and individuals with unspecific gastrointestinal symptoms [16]. Despite these associations, it remains unclear whether protozoa contribute to or are a consequence of shifts in gut microbial composition. While presence of protozoa is linked to bacterial composition, inflammatory factors in UC may further influence their persistence in the gut. As both protozoa are regarded as anaerobic organisms, [17] potential increased oxygen tension in inflamed ulcerative tissue, due to ubiquitous hyperemia and mucosal bleeding characteristic of UC, may limit their survival.

The observed association between lower disease scores and *D. fragilis* in our study was only significant in patients who tested microscopically positive for the eukaryote, although a similar trend was observed in PCR-positive cases. Real-time PCR, while highly sensitive, [18] may detect low levels of *D. fragilis* or non-viable organisms from previous infections. Microscopy provides direct evidence of parasite presence and may allow for some distinction between living and dead organisms. However, both techniques have limitations in assessing parasite viability and load. Although limited by small numbers, current results suggest that the load of an active infection, as shown by the load assessments and differences between results of microscopy and PCR, is associated with biochemical inflammation, as reflected by fecal calprotectin. However, this association was not reflected in significant correlations with clinical symptoms, as measured by SCCAI and Partial Mayo scores. A potential immune-modulatory role of *D. fragilis* remains largely unexplored, and further research is needed to investigate these associations.

Our findings, particularly the high prevalence of protozoa in asymptomatic healthy individuals, raise questions about the necessity of screening for these protozoa in donor selection for FMT. A pathogenic role of these protozoa remains debated in the literature. Present results in healthy individuals argue against this pathogenic role, suggesting that exclusion may even antagonize to some extent the selection of the most optimal donors. Moreover, in clinical IBD trials, including our own, *Blastocystis* spp. and *D. fragilis* are commonly used as exclusion criteria for patients undergoing investigational therapies. This exclusion is based on the rationale that potential confounding infections, especially those affecting stool frequency and abdominal pain, should be eliminated to ensure accurate assessment of the therapy being investigated. However, due to this exclusion, we were unable to gather additional endoscopic or histological data from UC patients with protozoa in our study, which could have further clarified the relationship between the presence of these protozoa and disease activity. Additionally, as our results suggest, by excluding *D. fragilis*, we may have inadvertently excluded patients with lower disease activity scores, potentially influencing clinical outcomes.

To our knowledge, this is the largest UC cohort to date in which *D. fragilis* prevalence has been investigated. Adding to previous reports on prevalence of *Blastocystis* spp. in UC, we also observed a lower prevalence of *D. fragilis* in UC patients. Furthermore, our findings indicate an inverse relationship between *D. fragilis* and clinical disease severity, as well as a negative association between *D. fragilis* load and fecal calprotectin. These results bring challenge the current practice of excluding donors and patients with evidence of *Blastocystis* spp. and *D. fragilis* in their feces from FMT and clinical IBD trials. Further research in larger cohorts is needed to confirm current findings, while basic research is required to clarify whether these protozoa play a role in UC pathogenesis or are a consequence of the disease, and explore their interactions with other microbial communities.

## Data Availability

No datasets were generated or analysed during the current study.
